# Longitudinal clinical, cognitive and biomarker profiles in dominantly inherited versus sporadic early-onset Alzheimer’s disease

**DOI:** 10.1093/braincomms/fcad280

**Published:** 2023-10-18

**Authors:** Jorge J Llibre-Guerra, Leonardo Iaccarino, Dean Coble, Lauren Edwards, Yan Li, Eric McDade, Amelia Strom, Brian Gordon, Nidhi Mundada, Suzanne E Schindler, Elena Tsoy, Yinjiao Ma, Ruijin Lu, Anne M Fagan, Tammie L S Benzinger, David Soleimani-Meigooni, Andrew J Aschenbrenner, Zachary Miller, Guoqiao Wang, Joel H Kramer, Jason Hassenstab, Howard J Rosen, John C Morris, Bruce L Miller, Chengjie Xiong, Richard J Perrin, Ricardo Allegri, Patricio Chrem, Ezequiel Surace, Sarah B Berman, Jasmeer Chhatwal, Colin L Masters, Martin R Farlow, Mathias Jucker, Johannes Levin, Nick C Fox, Gregory Day, Maria Luisa Gorno-Tempini, Adam L Boxer, Renaud La Joie, Gil D Rabinovici, Randall Bateman

**Affiliations:** Department of Neurology, Washington University in St Louis, St Louis, MO 63108, USA; Department of Neurology, UCSF Weill Institute for Neurosciences, University of California, San Francisco, San Francisco, CA 94158, USA; Division of Biostatistics, Washington University in St Louis, St Louis, MO 63108, USA; Department of Neurology, UCSF Weill Institute for Neurosciences, University of California, San Francisco, San Francisco, CA 94158, USA; Division of Biostatistics, Washington University in St Louis, St Louis, MO 63108, USA; Department of Neurology, Washington University in St Louis, St Louis, MO 63108, USA; Department of Neurology, UCSF Weill Institute for Neurosciences, University of California, San Francisco, San Francisco, CA 94158, USA; Malinckrodt Institute of Radiology, Washington University in St Louis, St Louis, MO 63108, USA; Department of Neurology, UCSF Weill Institute for Neurosciences, University of California, San Francisco, San Francisco, CA 94158, USA; Department of Neurology, Washington University in St Louis, St Louis, MO 63108, USA; Department of Neurology, UCSF Weill Institute for Neurosciences, University of California, San Francisco, San Francisco, CA 94158, USA; Division of Biostatistics, Washington University in St Louis, St Louis, MO 63108, USA; Division of Biostatistics, Washington University in St Louis, St Louis, MO 63108, USA; Department of Neurology, Washington University in St Louis, St Louis, MO 63108, USA; Malinckrodt Institute of Radiology, Washington University in St Louis, St Louis, MO 63108, USA; Department of Neurology, UCSF Weill Institute for Neurosciences, University of California, San Francisco, San Francisco, CA 94158, USA; Department of Neurology, Washington University in St Louis, St Louis, MO 63108, USA; Department of Neurology, UCSF Weill Institute for Neurosciences, University of California, San Francisco, San Francisco, CA 94158, USA; Division of Biostatistics, Washington University in St Louis, St Louis, MO 63108, USA; Department of Neurology, UCSF Weill Institute for Neurosciences, University of California, San Francisco, San Francisco, CA 94158, USA; Department of Neurology, Washington University in St Louis, St Louis, MO 63108, USA; Department of Neurology, UCSF Weill Institute for Neurosciences, University of California, San Francisco, San Francisco, CA 94158, USA; Department of Neurology, Washington University in St Louis, St Louis, MO 63108, USA; Department of Neurology, UCSF Weill Institute for Neurosciences, University of California, San Francisco, San Francisco, CA 94158, USA; Division of Biostatistics, Washington University in St Louis, St Louis, MO 63108, USA; Department of Neurology, Washington University in St Louis, St Louis, MO 63108, USA; Department of Pathology and Immunology, Washington University in St Louis, St. Louis, MO 63108, USA; Department of Cognitive Neurology, Institute for Neurological Research Fleni, Buenos Aires, Argentina; Department of Cognitive Neurology, Institute for Neurological Research Fleni, Buenos Aires, Argentina; Department of Cognitive Neurology, Institute for Neurological Research Fleni, Buenos Aires, Argentina; Department of Neurology, University of Pittsburgh, Pittsburgh, PA 15213, USA; Massachusetts General Hospital, Harvard Medical School, Boston, MA 02114, USA; Florey Institute, The University of Melbourne, Melbourne 3052, Australia; Neuroscience Center, Indiana University School of Medicine at Indianapolis, IN 46202, USA; DZNE-German Center for Neurodegenerative Diseases, Tübingen 72076, Germany; Hertie-Institute for Clinical Brain Research, University of Tübingen, Tübingen 72076, Germany; Department of Neurology, Ludwig-Maximilians-University, Munich 80539, Germany; German Center for Neurodegenerative Diseases, Munich 81377, Germany; Munich Cluster for Systems Neurology (SyNergy), Munich 81377, Germany; Dementia Research Centre, Department of Neurodegenerative Disease, University College London Institute of Neurology, London WC1N 3BG, UK; Department of Neurology, Mayo Clinic Florida, Jacksonville, FL 33224, USA; Department of Neurology, UCSF Weill Institute for Neurosciences, University of California, San Francisco, San Francisco, CA 94158, USA; Department of Neurology, UCSF Weill Institute for Neurosciences, University of California, San Francisco, San Francisco, CA 94158, USA; Department of Neurology, UCSF Weill Institute for Neurosciences, University of California, San Francisco, San Francisco, CA 94158, USA; Department of Neurology, UCSF Weill Institute for Neurosciences, University of California, San Francisco, San Francisco, CA 94158, USA; Department of Radiology and Biomedical Imaging, University of California, San Francisco, San Francisco, CA 94158, USA; Department of Neurology, Washington University in St Louis, St Louis, MO 63108, USA

**Keywords:** early-onset Alzheimer’s disease, sporadic, dominantly inherited

## Abstract

Approximately 5% of Alzheimer’s disease cases have an early age at onset (<65 years), with 5–10% of these cases attributed to dominantly inherited mutations and the remainder considered as sporadic. The extent to which dominantly inherited and sporadic early-onset Alzheimer’s disease overlap is unknown. In this study, we explored the clinical, cognitive and biomarker profiles of early-onset Alzheimer’s disease, focusing on commonalities and distinctions between dominantly inherited and sporadic cases. Our analysis included 117 participants with dominantly inherited Alzheimer’s disease enrolled in the Dominantly Inherited Alzheimer Network and 118 individuals with sporadic early-onset Alzheimer’s disease enrolled at the University of California San Francisco Alzheimer’s Disease Research Center. Baseline differences in clinical and biomarker profiles between both groups were compared using *t*-tests. Differences in the rates of decline were compared using linear mixed-effects models. Individuals with dominantly inherited Alzheimer’s disease exhibited an earlier age-at-symptom onset compared with the sporadic group [43.4 (SD ± 8.5) years versus 54.8 (SD ± 5.0) years, respectively, *P* < 0.001]. Sporadic cases showed a higher frequency of atypical clinical presentations relative to dominantly inherited (56.8% versus 8.5%, respectively) and a higher frequency of APOE-ε4 (50.0% versus 28.2%, *P* = 0.001). Compared with sporadic early onset, motor manifestations were higher in the dominantly inherited cohort [32.5% versus 16.9% at baseline (*P* = 0.006) and 46.1% versus 25.4% at last visit (*P* = 0.001)]. At baseline, the sporadic early-onset group performed worse on category fluency (*P* < 0.001), Trail Making Test Part B (*P* < 0.001) and digit span (*P* < 0.001). Longitudinally, both groups demonstrated similar rates of cognitive and functional decline in the early stages. After 10 years from symptom onset, dominantly inherited participants experienced a greater decline as measured by Clinical Dementia Rating Sum of Boxes [3.63 versus 1.82 points (*P* = 0.035)]. CSF amyloid beta-42 levels were comparable [244 (SD ± 39.3) pg/ml dominantly inherited versus 296 (SD ± 24.8) pg/ml sporadic early onset, *P* = 0.06]. CSF phosphorylated tau at threonine 181 levels were higher in the dominantly inherited Alzheimer’s disease cohort (87.3 versus 59.7 pg/ml, *P* = 0.005), but no significant differences were found for t-tau levels (*P* = 0.35). In summary, sporadic and inherited Alzheimer’s disease differed in baseline profiles; sporadic early onset is best distinguished from dominantly inherited by later age at onset, high frequency of atypical clinical presentations and worse executive performance at baseline. Despite these differences, shared pathways in longitudinal clinical decline and CSF biomarkers suggest potential common therapeutic targets for both populations, offering valuable insights for future research and clinical trial design.

## Teaser

Llibre-Guerra *et al*. show that dominantly inherited and sporadic early-onset Alzheimer’s disease differ in baseline clinical presentation and cognitive profiles. However, longitudinal cognitive decline and biomarker profiles are similar across both groups. These findings suggest shared pathways between dominantly inherited and sporadic early-onset Alzheimer’s disease.

## Graphical Abstract

**Graphical Abstract:**
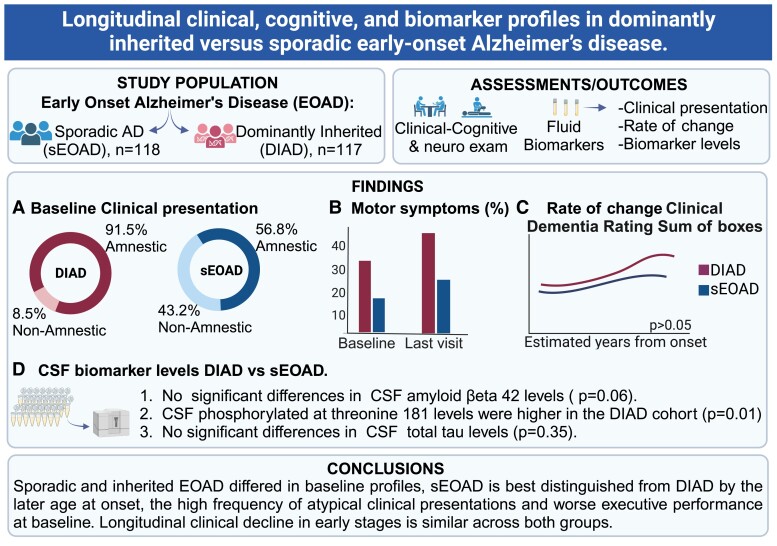

## Introduction

Alzheimer’s disease (AD) is the most common cause of dementia, with more than 131 million people worldwide expected to be affected by 2050.^1^ In 2019, the global economic burden of Alzheimer’s disease and related dementias was estimated at $2.8 trillion and is projected to increase to $16.9 trillion ($11.3 trillion–$27.3 trillion) in 2050.^2^ Low- and middle-income countries would account for 65% of the global economic burden in 2050, as compared with only 18% in 2019.^2,3^ Alzheimer’s disease dementia is also among the most costly illnesses in the USA, with an estimated yearly expenditure between $157 billion and $321 billion.^4^ Though generally considered a disease of the elderly, ∼5% of Alzheimer’s disease dementia cases have an early age at onset, which is defined as symptom onset before age 65 years.^5,6^ Patients with early-onset Alzheimer’s disease (EOAD) pose a clinical challenge and a scientific enigma.^7^ Alzheimer’s disease is particularly devastating when it occurs at younger ages, as it impacts individuals during a peak time of family, professional and financial responsibilities, leading to the loss of decades of life expectancy.^8,9^ About 5–10% of EOAD carry established dominantly inherited Alzheimer’s disease (DIAD) mutations in the presenilin 1 (*PSEN1*), presenilin 2 (*PSEN2*) or amyloid precursor protein (*APP*) genes leading to early and aggregation of amyloid β (Aβ).^10-13^ The remainder of patients who develop EOAD do not carry an established pathogenic mutation for Alzheimer’s disease and are therefore described as having ‘sporadic’ EOAD (sEOAD).^14^

Although accumulation of Aβ peptides is thought to be the common initiating event in Alzheimer’s disease, leading to the downstream spread of tau pathology, synaptic loss and neurodegeneration,^10,15^ there are reported clinical, cognitive and pathological differences between typical late-onset Alzheimer’s disease (LOAD), sEOAD and DIAD.^16,17^ Patients with EOAD show a more rapid clinical decline and shorter survival than LOAD patients.^18-20^ A high percentage of sEOAD cases (∼25–50%) present with non-amnestic presentations or atypical variants, such as the logopenic variant of primary progressive aphasia, posterior cortical atrophy and behavioural or dysexecutive variants of Alzheimer’s disease.^9,21,22^ The higher frequency of these atypical presentations in sEOAD suggests that a younger age of onset of Alzheimer’s disease may have different pathogenic drivers or selectively effect different neural networks compared to LOAD. However, these atypical Alzheimer’s disease presentations have been reported less frequently in DIAD,^23^ and patients carrying DIAD mutations tend to present with an amnestic syndrome similar to LOAD.^24^

Nevertheless, a great diversity of focal neurologic findings have been reported in DIAD, including visual agnosia, spastic paraplegia, ataxia, aphasia and behavioural changes.^16^ Post-mortem studies comparing the burden of Alzheimer’s disease pathology in early-onset and late-onset patients demonstrate a higher overall burden of neurofibrillary tangles (to a greater degree than neuritic plaques) and more severe neurodegeneration in younger patients,^25-34^ and similar results have been reported with tau PET and structural MRI when comparing DIAD with LOAD cohorts.^17,35,36^

Despite some evidence suggesting differences between sEOAD and DIAD, previous studies examined cross-sectional cohorts with no direct comparisons using similar measures.^7,35,37^ Therefore, the extent to which clinical presentations and cognitive and biomarker profiles in DIAD overlap with sEOAD remains unknown, and to what extent an earlier age of onset is the cause for atypical clinical phenotypes in sporadic versus DIAD remains to be determined.

To address this gap, we aimed to compare EOAD in two longitudinal observational studies: an early age-of-onset sporadic Alzheimer’s disease cohort followed at the University of California San Francisco Alzheimer’s Disease Research Center (UCSF ADRC) and the Dominantly Inherited Alzheimer Network (DIAN). The overall goal of the study was to compare the clinical presentation, cognitive performance and CSF biomarker concentrations in DIAD and sEOAD. A complementary comparison of PET molecular imaging biomarkers is reported in a separate manuscript (see Iaccarino *et al*., submitted). The results will expand our understanding of the relationships between clinical phenotype, cognitive decline and molecular pathology across different subtypes of Alzheimer’s disease.

## Materials and methods

### Participants

Existing data from two non-overlapping cohorts were used to retrospectively compare clinical presentations, cognitive performance and CSF biomarker profiles in DIAD and sEOAD. For both cohorts, only symptomatic participants with a global Clinical Dementia Rating® (CDR®) of >0 were included.^38^ Participants were classified as having sEOAD if they did not have a family history of dementia that followed an autosomal dominant pattern and tested negative for known mutations associated with DIAD. Participants with DIAD were all confirmed to have known pathogenic mutations that cause familial Alzheimer’s disease (see below). All participants in the study provided written informed consent or assent with proxy consent. The institutional review boards at DIAN participating sites and at UCSF approved all aspects of the study.

sEOAD participants were selected from ongoing longitudinal studies at the UCSF ADRC.

sEOAD participants were required to (i) have biomarker evidence of Alzheimer’s disease (positive amyloid PET scan or CSF biomarkers), (ii) have at least one clinical assessment (detailed neurological and neuropsychological examination), (iii) have age at reported symptoms onset <65 years old, (iv) absence of a family history of dementia that followed an autosomal dominant pattern and did not have evidence of a mutation associated with DIAD and (v) have a clinical diagnosis of mild cognitive impairment or dementia due to Alzheimer’s disease. sEOAD participants (*n* = 118) were subsequently divided into two groups according to clinical presentation: amnestic Alzheimer’s disease (*n* = 51) or non-amnestic Alzheimer’s disease [*n* = 67, including primary progressive aphasia^39^ (*n* = 34); posterior cortical atrophy^40^ (*n* = 22); and behavioural/dysexecutive variant Alzheimer’s disease^41^ (*n* = 11)]. For this study, the reported clinical diagnosis for each participant refers to the clinical syndromic diagnosis at the most recent UCSF clinical assessment.

DIAD participants were selected from the DIAN (DIAN data freeze 15) observational study.^42^ All participants in DIAN are recruited from families with DIAD pathogenic variants in *APP*, *PSEN1* or *PSEN2* genes. Participant enrolment and procedures have been previously described.^42,43^ For this study, only symptomatic (CDR > 0) mutation carriers with at least one clinical assessment (detailed neurological and neuropsychological examination) and biomarkers were included (*n* = 117). Asymptomatic (CDR = 0) DIAN non-mutation carriers were also included as an age-matched cognitively unimpaired control group (*n* = 168).

All participants underwent a comprehensive clinical assessment, cognitive testing and CSF and blood draws for biospecimen collection. Full details of participating sites, enrolment and assessments in DIAN and UCSF ADRC have been published.^42,44^ Measures relevant to this comparison are detailed below.

### Clinical and neuropsychological assessments

All participants underwent a comprehensive clinical assessment with the National Alzheimer’s Coordinating Center uniform data set.^45-47^ This assessment included informant interviews, personal medical history, a family history, physical and neurologic examination, functional assessments and cognitive testing. Data evaluated included the clinical diagnosis; demographic features; the age of symptom onset; first disease symptom; presence of cognitive, behavioural, neuropsychiatric [as measured by Neuropsychiatric Inventory—Questionnaire (NPI-Q)] and motor symptoms that developed throughout the disease; and neurological examination findings. For both cohorts, the participant’s estimated years from symptom onset (EYO) was defined as the participant’s age at baseline minus their age-at-symptom onset (AAO).^10,11,43^ The AAO was calculated based on the age at the first progressive symptom, as stated in the participant’s clinical history. Clinical dementia severity was determined with the global CDR® in accordance with standard protocols and criteria.^38,48^ Clinical syndromic diagnoses for typical and atypical Alzheimer’s disease syndromes were defined using the same clinical criteria in both cohorts, although consensus diagnosis was performed separately using cohort-specific procedures.

Core neuropsychological measures were drawn from the National Alzheimer’s Coordinating Center cognitive battery. Only neuropsychological measures that were common across both cohorts were included in the analysis. Clinical progression was assessed using consecutive scores on the Mini-Mental State Exam (MMSE) and CDR Sum of Boxes (CDR-SB). The rate of cognitive decline was estimated using individual measures of cognitive performance (e.g. digit symbol, category and verbal fluency, Trails A and B and logical memory delayed recall) and a composite score comprised of the MMSE, logical memory, digit symbol and animal fluency tests. Details of the DIAN cognitive composite and measurement properties have been published elsewhere.^49^

### Biochemical analysis

DNA was extracted from blood using standard protocols. In the DIAN cohort, the presence or absence of a DIAD mutation was determined using PCR-based amplification of the appropriate exon followed by Sanger sequencing methods.^50^ In the UCSF ADRC group, screening for pathogenic variants in the most common causative genes for Mendelian forms of Alzheimer’s disease and frontotemporal dementia (*MAPT*, *C9orf72*, *GRN*, *TARDBP*, *FUS*, *PSEN1*, *PSEN2* and *APP*) was performed as previously described.^51^  *APOE* genotyping was performed for both cohorts.^51,52^

Protocols for CSF collection and processing were consistent with the Alzheimer’s Disease Neuroimaging Initiative. CSF from both cohorts was collected in the morning under fasting conditions by lumbar puncture and immediately placed on dry ice. Samples from both cohorts were shipped on dry ice to the DIAN Biomarker Core laboratory at Washington University (St Louis, MO, USA). Samples were thawed and aliquoted into polypropylene tubes before storage at −80°C. Aβ1–42, total tau and phosphorylated tau at threonine 181 (p-tau181) were measured using the xMAP Luminex platform (INNO-BIA AlzBio3 for research-only reagents; Innogenetics) and according to standardized procedures.^53^ Both cohorts used the same lab procedures and assays but were run in different batches using different lot numbers. CSF biomarkers were available for 91/117 (77.8%) DIAD, 165/168 (98.2%) DIAN non-mutation carrier participants and 37/118 (31.2%) sEOAD.

### Statistical analysis

Demographic and baseline characteristics of the participants are summarized as mean ± SD for continuous variables and *n* (column percentage) for categorical variables. Group comparisons were performed using a two-sample *t*-test for continuous variables and *Z*-test for two proportions for categorical variables. Baseline cognitive performance was compared among groups after adjusting for baseline EYO, sex, years of education and *APOE*-*ε*4 status. The annual rate of change over the longitudinal follow-up period was estimated for each cohort (DIAD versus sEOAD) using random intercept and slope linear mixed-effects models and then plotted against baseline EYO to evaluate the trajectories of clinical and cognitive changes over the interval since symptom onset. Baseline EYO was included as a covariate, and all two-way interactions (cohort ∗ time, baseline EYO ∗ time and cohort ∗ baseline EYO) and a three-way interaction (cohort ∗ baseline EYO ∗ time) were tested. Sex, years of education and *APOE*-*ε*4 carrier status were included as covariates, and only significant effects were retained in the models. Interactions between *APOE*-*ε*4 carrier status (*ε*4+ versus *ε*4−), CDR® and group and between sex and group were not significant for any biomarkers, so were excluded from the final models. Statistical analyses were conducted with the PROC MIXED procedure in SAS software, version 9.4 (SAS Institute Inc., Cary, NC, USA). A *P*-value of <0.05 was considered to be statistically significant; given the exploratory nature of the study, results were not corrected for multiple comparisons.

## Results

### Subject characteristics

Of 235 participants with EOAD, 118 were considered sEOAD; 117 were determined to have a DIAD. Most DIAD participants were *PSEN1* mutation carriers [87 (74.4%)]; 9 (7.7%) were *PSEN2* mutation carriers, and 21 (17.9%) were *APP* mutation carriers. Demographics, baseline clinical presentation, co-morbidities and global cognitive measures from the DIAN and UCSF-sEOAD cohorts are presented in Table 1. Participants with DIAD were significantly younger at symptom onset than sEOAD [43.4 ± 8.5 years (mean ± standard deviation), range 21–64 versus 54.8 years (SD 5.0), range 34–64, *P* < 0.001]. We found significant age and education differences between DIAD and sEOAD (*P* < 0.001), but there were no significant differences in sex for these groups (*P* = 0.56). At baseline, DIAD and sEOAD participants were well matched for functional status, as measured by CDR-SB and MMSE; sEOAD participants had a longer duration as measured by EYO. Compared with sEOAD, motor manifestations (including parkinsonism, tremor, early falls and/or pyramidal signs) were higher in DIAD cohort [32.5% versus 16.9% at baseline (*P* = 0.006) and 46.1% versus 25.4% at last visit (*P* = 0.001); see Table 1]. Participants in the sEOAD cohort showed a high frequency of atypical presentations relative to DIAD carriers (56.8% versus 8.5%, respectively). Overall, DIAD participants had a lower frequency of APOE-ε4 relative to sEOAD (28.2% versus 50.0%, *P* = 0.001; Table 1). In a subset analysis, APOE-ε4 positivity was significantly more common in the amnestic sEOAD group than in the DIAD group (60.78% versus 28.21%, *P* = 0.001), but this difference was less prominent when contrasting non-amnestic sEOAD and DIAD (41.79% versus 28.21%, *P* = 0.06).

**Table 1:** Demographic and baseline characteristics: symptomatic DIAD versus sEOAD

Characteristic	DIAD *n* = 117	sEOAD *n* = 118	Significance level (*P*-value)
Age at onset, mean (SD)	43.4 (8.5)	54.8 (5.0)	**<0**.**001**
Age at baseline visit, mean (SD)	46.8 (9.2)	59.2 (5.0)	**<0**.**001**
Female, *n* (%)	62 (52.9)	67 (56.8)	0.56
Race/ethnicity			0.12
Non-Hispanic White	97 (87.4)	106 (89.8)	
Asian	6(5.4)	3(2.5)	
African American	1 (0.9)	3(2.5)	
Refuse to state/unknown	7(6.3)	5(3.2)	
Years of education, mean (SD)	13.6 (3.5)	16.3 (2.8)	**<0**.**001**
Symptoms duration*, mean (SD)	3.4 (2.7)	4.4 (1.8)	**0**.**001**
Hypertension, *n* (%)	14(11.9)	30 (25.4)	**<0**.**001**
Cardiovascular disease, *n* (%)	1(0.9)	2 (1.7)	0.16
Cerebrovascular disease, *n* (%)	1(0.9)	0	
Diabetes mellitus, *n* (%)	3(2.6)	3 (2.5)	0.08
Co-morbidity (2 or more), *n* (%)	2(1.7)	1 (0.8)	0.32
APOE-ε4(+), *n* (%)	33 (28.2)	59 (50.0)	**0**.**001**
*PSEN1*, *n* (%)	87 (74.4)	0	
*PSEN2*, *n* (%)	9 (7.7)	0	
*APP*, *n* (%)	21 (17.9)	0	
MMSE, mean (SD)	22.0 (6.9)	21.3 (5.7)	0.38
CDR, *n* (%)			
0.5	75 (64.1)	67 (56.8)	0.25
1	30 (25.6)	50 (42.4)	0.01
2/3	12 (10.2)	1 (0.85)	0.03
CDR-SB at baseline, mean (SD)	3.9 (3.9)	4.0 (1.9)	0.71
NPI-Q at baseline	8.3 (7.1)	6.1 (7.6)	0.02
GDS	3.9 (3.2) (*n* = 115)	3.4 (2.7) (*n* = 84)	0.18
Baseline motor Symptoms, *n* (%)	38 (32.5)	20 (16.9)	**0**.**01**
Last visit motor symptoms, *n* (%)	54 (46.2)	30 (25.4)	**<0**.**001**
Clinical Presentation			
Amnestic	107 (91.5)	51 (43.2)	**<0**.**001**
Non-Amnestic	10 (8.5)	67 (56.8)	

*APOE*-ε4(+) refers to presence of at least one ε4 allele of apolipoprotein E. Co-morbidity was defined as having two or more non-communicable disorders (e.g. diabetes mellitus and hypertension) or illnesses co-occurring in the same participant. *Symptom duration was defined as the time (years) from age at first progressive symptom to baseline assessment. Motor signs were considered to be present if evidence of parkinsonism, gait disorder, early falls, tremor and pyramidal signs. Significant differences are highlighted as bold values. APP, amyloid precursor protein; CDR, Clinical Dementia Rating Scale (scores range from 0 to 3, with higher scores indicating worse cognition and daily function); CDR-SB, Clinical Dementia Rating Scale Sum of Boxes (scores range from 0 to 18, with higher scores indicating worse cognition and daily function); GDS, Geriatric Depression Scale; NPI-Q, Neuropsychiatric Inventory Questionnaire; PSEN1, presenilin 1; PSEN2, presenilin 2.

### Baseline cognitive performance

The baseline cognitive assessments adjusting for baseline EYO, sex, years of education and APOE-ε4 status are shown in Table 2. At baseline, there were no significant differences in cognitive performance between the sEOAD and DIAD on logical memory (*P* = 0.74), letter fluency (*P* = 0.54) and naming (Boston Naming Test) (*P* = 0.42) (Table 2). Compared with DIAD, sEOAD had significantly lower scores in executive function/working memory at baseline (Table 2). Because of a higher frequency of atypical Alzheimer’s disease syndromes in the sEOAD, we divided the sEOAD according to typical (amnestic predominant syndrome) versus atypical presentations (non-amnestic predominant syndrome). After controlling for EYO, sex, education and APOE4 status. Comparisons of baseline cognitive performance in the sEOAD (amnestic and non-amnestic groups) versus DIAD groups are shown in Supplementary Table 1. Both amnestic and non-amnestic sEOAD participants performed significantly worse on digit span backwards (DIAD versus amnestic sEOAD, *P* = 0.001; DIAD versus non-amnestic sEOAD, *P* = 0.01), category fluency (DIAD versus amnestic sEOAD, *P* = 0.003; DIAD versus non-amnestic sEOAD, *P* = 0.001) and Trail Making Test Part B (DIAD versus amnestic sEOAD, *P* = 0.03; DIAD versus non-amnestic sEOAD, *P* < 0.001). In addition, the non-amnestic sEOAD showed worse cognitive performance on digit span forward (*P* = 0.01), Trail Making Test Part A and digit symbol substitution (*P* = 0.001).

**Table 2:** Baseline cognitive performance: symptomatic DIAD versus sEOAD

Characteristic	DIAD	sEOAD	Significance level (*P*-value*)
Logical memory (immediate recall), mean (SD), *n*	6.1 (4.6) *n* = 113	4.7 (4.3) *n* = 45	0.08
Logical memory (delayed recall), mean (SD), *n*	4.3 (4.5) *n* = 111	4.0 (4.3) *n* = 43	0.69
Category fluency (vegetables), mean (SD), *n*	9.3 (4.3) *n* = 101	6.7 (4.7) *n* = 43	**<0**.**001**
Category fluency (animals), mean (SD), *n*	15.3 (6.0) *n* = 113	10.5 (5.5) *n* = 101	**<0**.**001**
Letter fluency, mean (SD), *n*	10.6 (4.8) *n* = 99	11.8 (5.1) *n* = 35	0.54
Digit span forward, mean (SD), *n*	6.6 (2.5) *n* = 113	5.7 (2.4) *n* = 101	**<0**.**01**
Digit span backward, mean (SD), *n*	4.9 (2.3) *n* = 113	3.8 (2.2) *n* = 101	**0**.**001**
Trail Making Test Part A, mean (SD), *n*	54.9 (41.1) *n* = 106	81.6 (51.6) *n* = 66	**<0**.**001**
Trail Making Test Part B, mean (SD), *n*	147.7 (102.4) *n* = 86	207.8 (97.8) *n* = 47	**<0**.**001**
Boston Naming Test, mean (SD), *n*	23.6 (5.7) *n* = 106	22.7 (7.3) *n* = 43	0.27
Digit symbol, mean (SD), *n*	34.9 (19.1) *n* = 106	22.7 (13.9) *n* = 28	**<0**.**001**

Logical memory, category fluency, letter fluency, digit span, Boston Naming Test and digit symbol: lower scores indicating poorer cognitive performance. Trail Making Test scores: higher scores indicating poorer cognitive performance. *P-values were adjusted for baseline EYO, sex, education and APOE4 status. Significant differences are highlighted as bold values.

### Behavioural features

**Figure 1:**
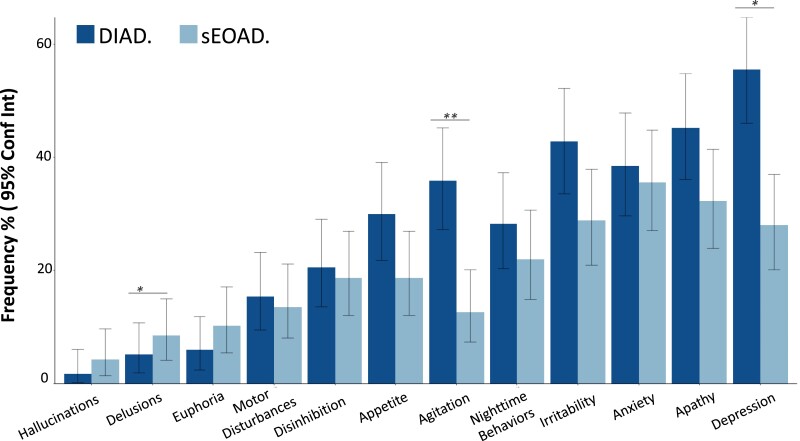
**NPI-Q: sEOAD versus DIAD.** (**A**) Percentage of respondents endorsing each item of the NPI-Q among DIAD (*n* = 117) and sEOAD (*n* = 118) cohorts. The *P*-values for comparing the two cohorts are calculated from Fisher’s exact test: *P = 0.01; **P = 0.001. Absolute number, percentage and associated *P*-values are shown in Supplementary Table 2. Conf Int, confidence interval.

At baseline assessment, DIAD participants had higher mean ratings on the NPI-Q relative to sEOAD [8.3 (7.1) versus 6.1 (7.7), *P* = 0.02] (Table 1). The four most prevalent neuropsychiatric symptoms in both cohorts included depression, irritability, apathy and anxiety (Fig. 1). When examining individual items from the NPI-Q, we found that the frequencies of agitation and depression were higher in DIAD (35.9% versus 12.7%, *P* = 0.001 and 55.6% versus 27.9%, *P* = 0.01, respectively). Delusions had a higher frequency in sEOAD (*P* = 0.045; Fig. 1 and Supplementary Table 2). Differences in NPI scores between DIAD and sEOAD remained significant after controlling for CDR and EYO. No other items differed between DIAD and sEOAD. Longitudinal trajectories of specific NPI domains did not differ between groups (Table 3).

**Table 3:** Estimated annual rate of change (standard error): symptomatic DIAD versus sEOAD by baseline EYO = 1, 5 and 10

		DIAD		sEOAD		
	EYO	Rate of change	*P*-value*	Rate of change	*P*-value*	*P*-value* comparing DIAD versus sEOAD
Cognitive Composite	**1**	−0.20 (0.06)	<0.001	−0.31 (0.10)	<0.01	0.32
	**5**	−0.35 (0.07)	<0.001	−0.28 (0.07)	<0.001	0.37
	**10**	−0.54 (0.16)	<0.001	−0.24 (0.16)	0.15	0.17
CDR-SB, mean (SE)	**1**	0.89 (0.29)	0.003	1.36 (0.33)	<0.001	0.25
	**5**	2.11 (0.32)	<0.001	1.57 (0.23)	<0.001	0.11
	**10**	3.63 (0.71)	<0.001	1.82 (0.54)	<0.001	**0**.**03**
MMSE mean (SE)	**1**	−1.30 (0.48)	0.008	−3.66 (0.60)	<0.001	**<0**.**01**
	**5**	−3.03 (0.56)	<0.001	−2.77 (0.42)	<0.001	0.67
	**10**	−5.18 (1.24)	<0.001	−1.67 (0.97)	0.09	**0**.**02**
NPI-Q mean (SE)	**1**	0.33 (0.83)	0.69	1.32 (0.90)	0.15	0.39
	**5**	1.62 (1.03)	0.12	0.72 (0.67)	0.29	0.41
	**10**	3.22 (2.33)	0.17	−0.02 (1.64)	0.99	0.24
Category fluency (animals), mean (SE)	**1**	−0.93 (0.49)	0.06	−1.19 (0.88)	0.18	0.78
	**5**	−1.84 (0.65)	0.01	−0.77 (0.60)	0.19	0.15
	**10**	−2.98 (1.44)	0.04	−0.25 (1.44)	0.86	0.16
Category fluency (vegetable), mean (SE)	**1**	−0.62 (0.37)	0.11	−0.62 (0.75)	0.41	0.99
	**5**	−1.40 (0.51)	0.01	−1.65 (0.49)	0.001	0.66
	**10**	−2.37 (1.11)	0.03	−2.95 (1.13)	0.01	0.71
Letter fluency, mean (SE)	**1**	−0.32 (0.37)	0.40	2.63 (1.33)	0.052	**0**.**03**
	**5**	−1.09 (0.56)	0.06	−1.41 (0.82)	0.09	0.71
	**10**	−2.05 (1.21)	0.09	−6.45 (2.29)	0.01	0.08
Logical memory (immediate recall), mean (SE)	**1**	−0.52 (0.32)	0.11	−0.37 (0.82)	0.65	0.85
	**5**	−1.00 (0.44)	0.02	−1.41 (0.53)	0.01	0.49
	**10**	−1.60 (0.94)	0.09	−2.71 (1.40)	0.06	0.50
Logical memory (delayed recall), mean (SE)	**1**	−0.17 (0.28)	0.55	−0.05 (0.73)	0.95	0.87
	**5**	−0.76 (0.39)	0.05	−1.84 (0.49)	<0.001	0.05
	**10**	−1.50 (0.84)	0.08	−4.09 (1.28)	<0.001	0.089
Digit symbol, mean (SE)	**1**	−4.32 (1.37)	<0.001	−5.97 (4.36)	0.18	0.70
	**5**	−7.18 (1.89)	<0.001	−4.19 (2.80)	0.14	0.32
	**10**	−10.75 (4.04)	<0.001	−1.96 (7.01)	0.78	0.28
Digit span forward, mean (SE)	**1**	−0.37 (0.19)	0.06	−0.51 (0.35)	0.15	0.69
	**5**	−1.48 (0.25)	<0.001	−0.62 (0.23)	0.01	**0**.**004**
	**10**	−2.87 (0.56)	<0.001	−0.76 (0.57)	0.18	**0**.**01**
Digit span backward, mean (SE)	**1**	−0.30 (0.14)	0.04	−0.08 (0.26)	0.77	0.17
	**5**	−0.30 (0.21)	0.17	−0.77 (0.19)	<0.001	0.054
	**10**	−0.29 (0.48)	0.54	−1.83 (0.47)	<0.001	**0**.**02**
Trail Making Test Part A, mean (SE)	**1**	6.80 (3.81)	0.081	2.72 (9.58)	0.78	0.68
	**5**	22.00 (4.99)	<0.001	10.48 (5.74)	0.07	0.07
	**10**	41.00 (11.02)	<0.001	20.18 (14.67)	0.17	0.24
Trail Making Test Part B, mean (SE)	**1**	17.55 (9.12)	0.06	−41.65 (25.77)	0.11	**0**.**03**
	**5**	21.77 (15.70)	0.17	26.81 (16.62)	0.11	0.78
	**10**	27.05 (35.80)	0.45	112.39 (41.84)	0.01	0.11
Boston Naming Test, mean (SE)	**1**	−0.79 (0.55)	0.15	−1.72 (1.49)	0.25	0.54
	**5**	−2.11 (0.68)	<0.001	−2.74 (0.86)	<0.01	0.51
	**10**	−3.75 (1.45)	0.01	−4.01 (2.29)	0.08	0.92

Logical memory, category fluency, letter fluency, digit span, Boston Naming Test and digit symbol: lower scores indicating poorer cognitive performance. Trail Making Test scores: higher scores indicating poorer cognitive performance. Cognitive composite is the mean of the standardized scores for animal naming, delayed recall, digit symbol and MMSE tests. *P-values were adjusted for baseline EYO, sex, education and APOE4 status. Significant differences are highlighted as bold values.

### Longitudinal functional and cognitive rate of decline

**Figure 2:**
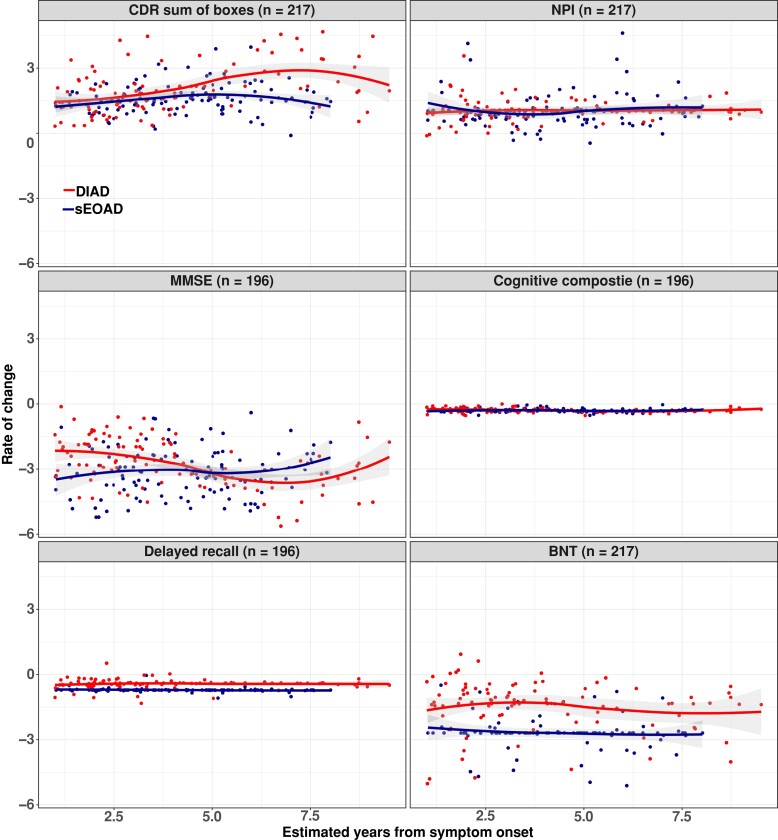
**Estimated mean rate of change from baseline with standard error for DIAD and sEOAD groups.** (**A**) CDR-SB scores range from 0 to 18, with higher scores indicating worse cognition and daily function (DIAD versus sEOAD, *P* = NS; 0.04 at EYO = 10). (**B**) NPI-Q with higher scores indicating high burden of neuropsychiatric symptoms (DIAD versus sEOAD, *P* = NS). (**C**) MMSE with lower scores indicating worse cognition (DIAD versus sEOAD, *P* = <0.05; NS at EYO = 5). (**D**) Global cognitive composite, with lower scores indicating worse cognition (DIAD versus sEOAD, *P* = NS). (**E**) Logical memory: logical memory delayed recall, scores range from 0 to 25, with lower scores indicating poorer cognitive performance (DIAD versus sEOAD, *P* = NS). (**F**) Boston Naming Test (BNT) with lower scores indicating poorer cognitive performance (DIAD versus sEOAD, *P* = NS). Observed values from DIAD and sEOAD cohorts were represented by red and blue dots, respectively. The temporal patterns of rate of change were shown by locally estimated scatter smoothing curves. Random intercept random slope mixed-effects models were fitted to compare the difference between DIAD and sDOAD cohorts after adjusting for baseline EYO, sex, education and APOE4 status. Mean values and corresponding coefficient’s Wald *t*-test *P*-values. Mean values and corresponding coefficient’s Wald *t*-test *P*-values are shown in Table 3. Mean values for DIAD versus sEOAD amnestic and sEOAD non-amnestic are shown Table S1. NS, not significant.

Rates of longitudinal functional and cognitive decline across groups are shown in Table 3. Using a cognitive composite (MMSE, logical memory, digit symbol and animal fluency), the rate of cognitive decline was similar among the sEOAD and DIAD participants. The mean annual rates of change in the cognitive composite score at every EYO point for the sEOAD and DIAD cohorts, respectively, were for baseline EYO +1, −0.21 (0.04) versus −0.30 (0.09) points (*P* = 0.34); for baseline EYO +5 −0.35 (0.05) versus −0.30 (0.05) points (*P* = 0.46); and for baseline EYO +10 −0.53 (0.12) versus −0.30 (0.15) points (*P* = 0.23). No significant difference in rate of disease progression over time was detected for other clinical/cognitive measures including NPI-Q, category fluency (animal naming) or logical memory (see Fig. 2 and Table 3). After EYO = +10, the DIAD cohort showed faster functional decline as measured by CDR-SB [3.63 (0.71) versus 1.82 (0.54) points (*P* = 0.035)]. For MMSE score, the sEOAD showed faster progression at EYO +1 [−3.66 (0.60) versus −1.30 (0.48) points (*P* = 0.002)], while the DIAD cohort showed faster progression after EYO +10 [−5.18 (1.24) versus −1.67 (0.97) points (*P* = 0.02)]. Longitudinal rate of change and cohort differences on individual cognitive measures are shown in Table 3. Annual rates of change in the sEOAD amnestic and non-amnestic groups versus DIAD are shown in Supplementary Table 3.

### CSF biomarker profiles

**Figure 3:**
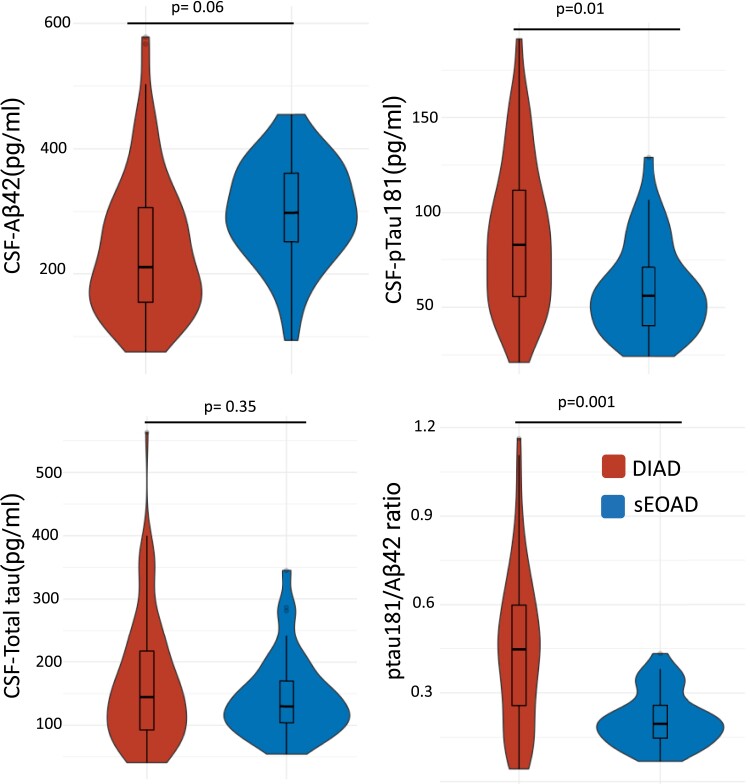
**CSF biomarkers of amyloid, tau and phosphorylated tau at threonine 181.** Biomarkers included (**A**) Aβ42, (**B**) p-tau181, (**C**) total tau and (**D**) pTau181/Aβ42. The central horizontal bar shows the median value, and the lower and upper boundaries show the SD. DIAD (*n* = 91); sEOAD (*n* = 37). Absolute mean differences, SD and associated *P*-values of ANOVA *F*-test are shown in Supplementary Table 4. *P*-value adjusted for CDR, age and APOEe4 status. Both cohorts used the same lab procedures and assays but were run in different batches using different lot numbers.

DIAD and sEOAD biomarker patterns were consistent with the presence of Alzheimer’s disease pathology, including reductions in Aβ42 (*P* < 0.0001) and increases in p-tau181 (*P* < 0.0001) compared with the DIAN non-carrier group (Supplementary Table 4). After adjusting for CDR, age and APOE*-*ε4 status, there was a non-significant trend for lower CSF Aβ42 levels in DIAD than in sEOAD (243 ± 116 pg/ml versus 296 ± 82 pg/ml, *P* = 0.06; see Fig. 3). CSF p-tau181 levels were higher in the DIAD cohort (87.3 ± 39.3 pg/ml versus 59.7 ± 24.8 pg/ml, *P* = 0.01), while no significant differences were found for t-tau levels (*P* = 0.35). DIAD participants showed a higher pTau-181/Aβ42 ratio relative to sEOAD (0.4 ± 0.3 pg/ml versus 0.2 ± 0.1 pg/ml, *P* = 0.001).

## Discussion

The objective of this study was to better understand clinical profiles at presentation and disease progression among autosomal dominant versus sporadic forms of EOAD. Although previous studies have reported on clinical, cognitive and CSF biomarkers profiles in sEOAD and DIAD, to the best of our knowledge, this is the first study to directly compare individuals with sEOAD and those with DIAD using a similar methodology. Our sample of sEOAD and DIAD participants showed similar baseline global and memory impairment, rates of behavioural and cognitive decline and baseline neurodegeneration as measured by CSF total tau. Despite many similarities, we also observed several important differences. At baseline, sEOAD showed a higher frequency of non-amnestic presentations while DIAD showed a higher frequency of motor symptoms. Even within the amnestic group, sEOAD participants presented with lower performance on tests of executive function compared with DIAD. Conversely, the DIAD cohort showed a higher frequency of agitation and depression relative to sEOAD. Finally, participants with DIAD showed overall higher concentrations of CSF p-tau181 and a trend for lower CSF Aβ42 levels. The observed differences may shed light on potential differences in Aβ42/pTau metabolism leading to differences in neurofibrillary tangles distribution, neurodegeneration and possibly selective vulnerability that may explain differences in clinical presentation.

As previously reported, DIAD participants had an earlier age-at-symptom onset,^11^ which also may explain the lower frequency of medical co-morbidities relative to the sEOAD. Of note, age-at-symptom onset is determined by the clinician according to the family/caregiver report, which may lead to a higher ascertainment bias in sEOAD relative to DIAD; families with known mutations may seek diagnosis and treatment sooner due to knowledge of the disease and provide more accurate estimates of age-at-symptom onset.^54^ In addition, DIAD families are closely monitored prospectively before and after parental EYO leading to very accurate estimates of age-at-symptom onset. Conversely, patients with sEOAD are known to often face delays in diagnosis and to have early symptoms mistakenly attributed to non-neurologic causes (e.g. depression and hormonal changes).^55,56^ Better estimation of the age-at-symptom onset in DIAD relative to sEOAD may also explain the apparent shorter duration of the disease in DIAD.

Our findings showed a more heterogeneous clinical presentation in the sEOAD cohort relative to DIAD, which included a higher percentage of non-amnestic cognitive syndromes and neuropsychiatric symptoms. Beyond isolated case reports, patients with DIAD typically do not present with atypical clinical Alzheimer’s disease phenotypes,^23,24^ and the overwhelming majority of DIAD cases present as a primarily amnestic syndrome or a multi-domain syndrome including memory impairment.^16,57,58^ The mechanisms that drive phenotypic heterogeneity in sEOAD are not well understood. Clinical phenotypes in sEOAD are linked to differential patterns of neurofibrillary tangles, neurodegeneration and network dysfunction,^34,59^ with logopenic variant of primary progressive aphasia showing greater involvement of left hemisphere language networks, posterior cortical atrophy showing greater occipital and visual network involvement and dysexecutive/behavioural variants showing variable dysfunction of frontal and parietal networks. Some data suggest that neurodevelopmental language disorders may predispose to logopenic variant of primary progressive aphasia, while disorders of spatial reasoning may be associated with posterior cortical atrophy.^60^ It is also possible that different brain regions may have greater susceptibility to the mechanisms posited to trigger Aβ plaque accumulation, i.e. overproduction of Aβ peptides in DIAD versus reduced clearance of Aβ in sporadic Alzheimer’s disease.^61-63^ Differences in genetic risk factors in sEOAD may also explain higher susceptibility to non-amnestic phenotypes.^64,65^ Our results suggest that age alone is insufficient in explaining this aspect of clinical heterogeneity. Both cohorts showed a lower frequency of APOE-ε4 (<50%) than reported in most LOAD studies, suggesting that APOE-ε4 alone is unlikely a major driver for sEOAD or DIAD.

Patients with sEOAD showed worse performance at baseline relative to DIAD in several cognitive domains including executive function, visuospatial and language. Differences in cognitive performance at baseline were attenuated when comparing DIAD with the amnestic sEOAD group; however, lower performances on executive function remained. Longitudinally, both groups declined at similar rates, especially during the early stages of the disease; however, the DIAD group showed a faster decline in some measures during more advanced stages (e.g. CDR-SB, MMSE and digit span backward). Notably, the small sample sizes in these stages of the disease limit the interpretability of these results. There are several factors that may explain observed differences in cognitive performance between both groups. First, sEOAD is more heterogenous (even those with amnestic presentation), so more variability might be present in cognitive performance at presentation. Second, the estimation of age at onset and symptom duration in sEOAD is more prone to recall bias relative to DIAD, so our study might be capturing more advanced sEOAD individuals at baseline. Third, DIAD participants are on average younger and therefore may have better resilience mechanisms to cope with pathology at early disease stages, which may also explain the faster decline at later stages when resilience mechanisms are fully depleted.

Our findings suggest that, at baseline, the DIAD cohort presents with a higher frequency of depression and agitation, while sEOAD showed a higher frequency of delusions at baseline. Several DIAN subjects and family members in the study know their genetic status or are aware of at least a 50% risk of being mutation carriers, which could influence their affective state, especially around symptom onset.^66^ Alternatively, previous studies have shown that different patterns of neuropsychiatric symptoms across the Alzheimer’s disease spectrum may result from differences in Alzheimer’s disease pathology distribution in subcortical regions, suggesting the presence of a pattern of selective vulnerability extending to subcortical structures.^67^

In general, motor impairment at baseline was relatively frequent (>15%) in our cohort, especially in the DIAD group, where motor impairment was twice as common in DIAD compared with sEOAD. Motor presentations have been described in several *PSEN-1* pathogenic variants both as a presenting feature (spastic paraparesis) and with disease progression. Motor features in DIAD have been linked to specific mutations and greater involvement of the basal ganglia.^68-70^ Evidence of amyloid–PET binding in basal ganglia is known to be present in early phases of the diseases in DIAD,^71-74^ while sporadic Alzheimer’s disease is typically only described during more advanced stages of the disease.^75-77^

Despite differences in clinical presentation and cognitive performance at baseline between DIAD and sEOAD, the longitudinal rate of decline, CSF Aβ42 and neurodegeneration markers, as measured by total tau, were similar among groups. The similar rate of decline and the comparability of the CSF biomarkers suggest a common pathophysiology leading to disease onset and progression in the two groups. Comparison of amyloid and tau PET patterns in DIAD relative to sEOAD is underway (see Iaccarino L *et al*.).

The present study has several strengths, including the use of two well-characterized cohorts of EOAD, who had the same basic clinical and cognitive assessment, with biomarker-supported diagnoses of Alzheimer’s disease. However, the study is also subject to several limitations that may affect the interpretation and generalizability of results. First, this is a retrospective analysis, and although similar cognitive measures were used across both cohorts, each group was assessed at independent centres. Previous studies have shown that cross-cohort administration, scoring and procedural differences are frequent and may impact data interpretation.^78^ Furthermore, DIAN is a multi-site study including US and non-US centres, while the UCSF ADRC is a single site in the USA; future comparisons between DIAN and sEOAD multi-site studies (e.g. the multi-site Longitudinal Early-Onset AD Study)^79^ may help to overcome such limitations.^80^ Second, our study included a relatively high frequency of non-amnestic presentation in the sEOAD cohort; the clinical and research focus at UCSF on understanding atypical Alzheimer’s disease presentation may have resulted in a larger proportion of non-amnestic presentations than evident in the larger community, though higher rates of non-amnestic presentations in sEOAD have been reported at many centers.^81^ Despite the high frequency of non-amnestic presentation, we were underpowered to investigate the sporadic non-amnestic Alzheimer’s disease variants as independent groups, and our analysis included these variants as a single cohort. Each non-amnestic variant has distinct clinical and anatomical features, so the aggregation of these groups may obscure the detection of differences in the rate of cognitive decline or changes in CSF biomarkers. Similarly, we investigated all DIAD mutations as a single group, despite known clinical differences across different mutations. In addition, both cohorts included a limited sample size in the late stages of the disease, which limited the interpretability of the results during advanced clinical stages. Third, the relatively low number of sEOAD participants with available CSF samples and lack of CSF longitudinal assessments may limit our statistical power. In addition, although CSF samples from both cohorts were run in the same lab and using the same assay, each cohort was run in different batches using different lot numbers, which may create differences in the measurements of analytes. In addition, other Alzheimer’s disease relevant biomarkers including tau isoforms, neurofilament light and glial fibrillary acidic protein were not available for both cohorts at the time of the analysis. Future studies should further explore this question by comparing both cohorts using the same assays and lot numbers run together for direct comparison. Finally, both cohorts included in this study were predominantly non-Hispanic Whites with little to no representation of more diverse racial and ethnic groups; therefore, the generatability of our findings is limited and future studies should include more diverse groups.

In conclusion, sEOAD is best distinguished from DIAD by the later age at onset, the high frequency of atypical clinical presentations and worse executive performance at baseline, while a higher frequency of depression, agitation and motor signs is observed in DIAD. Despite the differences in clinical presentation and in underlying causes for Alzheimer’s disease development, both groups show similar disease progression, cognitive decline rate and CSF Aβ42 and total tau biomarker patterns, reflecting a common cascade of events leading to the emergence of symptomatic Alzheimer’s disease. Notably, after 10 years from onset, the DIAD group showed a faster decline in CDR-SB and MMSE. Further work is needed to better delineate common and distinct pathways that drive different subtypes of Alzheimer’s disease that may impact future therapeutic approaches. Our findings of similar patterns of longitudinal cognitive decline between DIAD and sEOAD suggest that we may be able to extrapolate models from DIAD into sEOAD. These findings indicate that despite having different causes, familial and sEOAD share many aspects of pathophysiology (e.g. biomarkers levels) and clinical progression. Differences in presenting clinical profiles suggest that clinical outcome measures tailored to each population may be needed in clinical trials specific to DIAD and sEOAD. Given both similarities and differences in biomarkers and underlying mechanisms, it is critical to the effectiveness of novel drugs in patients with DIAD and sEOAD.

## Supplementary Material

fcad280_Supplementary_Data

## Data Availability

Data supporting the findings of this study are available on request and will follow the policies of the DIAN (https://dian.wustl.edu) and of the UCSF ADRC (https://memory.ucsf.edu/research-trials/professional/open-science#Data-Sharing), both of which comply with the guidelines established by the Collaboration for Alzheimer's Prevention. Data are not publicly available in order to preserve the privacy of research participants.
