# Supplementary material for: Longitudinal clinical, cognitive and biomarker profiles in dominantly inherited versus sporadic early-onset Alzheimer’s disease
Source: Brain Commun. 2023 Oct 18;5(6):fcad280. doi: 10.1093/braincomms/fcad280 (PMC10629466; doi:10.1093/braincomms/fcad280)
Supplement: fcad280_Supplementary_Data [file fcad280_supplementary_data.docx]

# Supplementary material: Longitudinal clinical, cognitive, and biomarker profiles in dominantly inherited versus sporadic early-onset Alzheimer’s disease.

Jorge J Llibre-Guerra^1^**^†^**, Leonardo Iaccarino^2^**^†^**, Dean Coble^3^, Lauren Edwards^2^, Yan Li^3^, Eric McDade^1^, Amelia Strom^2^, Brian Gordon^4^, Nidhi Mundada^2^, Suzanne E. Schindler^1^, Elena Tsoy^2^, Yinjiao Ma^3^, Ruijin Lu^3^, Anne M Fagan^1^, Tammie L. S. Benzinger^4^, David Soleimani-Meigooni^2^, Andrew J. Aschenbrenner^1^, Zachary Miller^2^, Guoqiao Wang^3^, Joel H. Kramer^2^, Jason Hassenstab^1^, Howard J. Rosen^2^, John C. Morris^1^, Bruce L Miller^2^, Chengjie Xiong^3^, Richard J. Perrin^1,5^, Ricardo Allegri^6^, Patricio Chrem^6^, Ezequiel Surace^6^, Sarah B. Berman^7^, Jasmeer Chhatwal^8^, Colin L Masters^9^, Martin R. Farlow^10^, Mathias Jucker^11,12^, Johannes Levin^13,14,15^, Nick C. Fox^16^, Gregory Day^17^, Maria Luisa Gorno-Tempeni^2^, Adam L. Boxer^2^, Renaud La Joie^2^, Gil D. Rabinovici^2,18*^, Randall Bateman^1*^.

**^†^These authors contributed equally to this work.**

**Author affiliations:**

1. Department of Neurology, Washington University in St Louis, St Louis, MO, USA
2. Department of Neurology, UCSF Weill Institute for Neurosciences, University of California, San Francisco, San Francisco, CA, USA.
3. Division of Biostatistics, Washington University in St Louis, St Louis, MO, USA
4. Malinckrodt Institute of Radiology, Washington University in St Louis, St Louis, MO, USA
5. Department of Pathology and Immunology, Washington University in St Louis, St. Louis, MO, USA
6. Department of Cognitive Neurology, Institute for Neurological Research Fleni, Buenos Aires, Argentina.
7. Department of Neurology, University of Pittsburgh, Pittsburgh, PA USA
8. Massachusetts General Hospital, Harvard Medical School, Boston, MA
9. Florey Institute and The University of Melbourne, Melbourne, Australia.
10. Neuroscience Center. Indiana University School of Medicine at Indianapolis, Indiana
11. DZNE-German Center for Neurodegenerative Diseases, Tübingen, Germany.
12. Hertie-Institute for Clinical Brain Research, University of Tübingen, Tübingen, Germany.
13. Department of Neurology, Ludwig-Maximilians-University, Munich, Germany.
14. German Center for Neurodegenerative Diseases, site Munich.
15. Munich Cluster for Systems Neurology (SyNergy), Munich, Germany
16. Dementia Research Centre, Department of Neurodegenerative Disease, University College London Institute of Neurology, London, UK
17. Department of Neurology, Mayo Clinic Florida, Jacksonville, FL, USA.
18. Department of Radiology and Biomedical Imaging. University of California, San Francisco, San Francisco, CA, USA.

**Supplementary Table 1. ﻿Baseline Cognitive performance: dominantly inherited Alzheimer’s disease (DIAD) versus sporadic Early-Onset Alzheimer’s Disease (amnestic and non-amnestic).**

| **﻿Characteristic** | **DIAD** | **sEOAD**  **Amnestic** | **sEOAD**  **Non-Amnestic** | **Significance Level**  **(p value)** |
| --- | --- | --- | --- | --- |
| Logical Memory (Immediate Recall), mean (SD) N | 6.1 (4.6)  N=113 | 4.9 (4.5)  N=19 | 4.6923 (4.3)  N=26 | 0.27^1^, 0.10^2^, 0.75^3^ |
| Logical Memory (Delayed Recall), mean (SD) N | 4.3 (4.5)  N=111 | 3.7 (4.3)  N=19 | 4.2 (4.4)  N=24 | 0.79^1^, 0.72^2^, 0.96^3^ |
| Category Fluency (Vegetables), mean (SD) N | 9.3 (4.3)  N=101 | 6.5 (4.2)  N=38 | 6.9 (4.9)  N=56 | **0.003^1^, 0.01^2^**, 0.54^3^ |
| Category Fluency (Animals), mean (SD) N | 15.3 (6.0)  N=113 | 9.8 (5.0)  N=42 | 10.9 (5.9)  N=59 | **<.0001^1^, <.0001^2^**, 0.39^3^ |
| Letter Fluency, mean (SD) N | 10.6 (4.8)  N=99 | 10.4 (5.3)  N=13 | 12.3 (5.0)  N=22 | 0.89^1^, 0.34^2^, 0.42^3^ |
| Digit Span Forward, mean (SD) N | 6.6 (2.5)  N=113 | 5.9 (2.4)  N=42 | 5.5 (2.5)  N=59 | 0.07^1^, **0.002^2^**, 0.29^3^ |
| Digit Span Backward, mean (SD) N | 4.9 (2.3)  N=113 | 3.4 (2.3)  N=42 | 4.1 (2.2)  N=59 | **0.001^1^, 0.01^2^,** 0.3048^3^ |
| Trailmaking Test Part A, mean (SD) N | 54.9 (41.1)  N=106 | 72.9 (49.5)  N=23 | 86.3 (52.6)  N=43 | **0.02^1^, <.0001^2^**, 0.15^3^ |
| Trailmaking Test Part B, mean (SD) N | 147.7 (102.4)  N=86 | 205.1 (102.7)  N=18 | 209.5 (96.5)  N=29 | **0.03^1^, <.0001^2^**, 0.07^3^ |
| Boston Naming Test, mean (SD) N | 23.6 (5.7)  N=106 | 24.1 (6.5)  N=17 | 21.9 (7.8)  N=26 | 0.92^1^, 0.10^2^, 0.19^3^ |
| Digit Symbol, mean (SD) N | 34.9 (19.1)  N=106 | 23.8 (16.9)  N=10 | 22.2 (12.5)  N=18 | **0.04^1^, 0.001^2^**, 0.65^3^ |

Logical Memory, Category Fluency, Letter Fluency, Digit Span, Boston Naming Test and Digit Symbol: lower scores indicating poorer cognitive performance. Trail making Test scores: higher scores indicating poorer cognitive performance. WAIS, and MMSE tests. Superscripts for p-values: ^1^DIAN vs EOAM; ^2^DIAN vs EONA, ^3^EOAM vs EONA.

**Supplementary Table 2. Frequency of neuropsychiatric symptom dominantly inherited Alzheimer’s disease (DIAD) versus sporadic Early-Onset Alzheimer’s Disease.**

| **NPI symptoms** | **DIAD n(%)** | **CI, 95%** | **sEOAD n(%)** | **CI, 95%** | **Significance Level**  **(p value)** |
| --- | --- | --- | --- | --- | --- |
| Agitation** | 42 (35.9) | 27.2-45.3 | 15 (12.7) | 7.3-20.1 | **0.001** |
| Depression* | 65 (55.6) | 46.1-64.7 | 33 (28.0) | 20.1-37 | **0.01** |
| Anxiety | 45 (38.5) | 29.6-47.9 | 42 (35.6) | 27-44.9 | 0.80 |
| Apathy | 53 (45.3) | 36.1-54.8 | 38 (32.2) | 23.9-41.4 | 0.85 |
| Euphoria | 7 (6.0) | 2.4-11.9 | 12 (10.2) | 5.4-17.1 | 0.07 |
| Disinhibition | 24 (20.5) | 13.6-29 | 22 (18.6) | 12.1-26.9 | 0.33 |
| Irritability | 50 (42.7) | 33.6-52.2 | 34 (28.8) | 20.9-37.9 | 0.85 |
| Motor Disturbances | 18 (15.4) | 9.4-23.2 | 16 (13.6) | 8-21.1 | 0.89 |
| Night time Behaviors | 33 (28.2) | 20.3-37.3 | 26 (22.0) | 14.9-30.6 | 0.92 |
| Hallucinations | 2 (1.7) | 0.2-6 | 5 (4.2) | 1.4-9.6 | 0.18 |
| Delusions* | 6 (5.1) | 1.9-10.8 | 10 (8.5) | 4.1-15 | 0.045 |
| Appetite | 35 (29.9) | 21.8-39.1 | 22 (18.6) | 12.1-26.9 | 0.61 |

**CI: Confidence Interval**

**Supplementary Table 3. Estimated annual rate of change (standard error): dominantly inherited Alzheimer’s disease (DIAD) versus sporadic Early-Onset Alzheimer’s Disease (amnestic and non-amnestic).**

|  |  | **DIAD** | | **sEOAD – Amnestic** | | **sEOAD – non-Amnestic** | |  |
| --- | --- | --- | --- | --- | --- | --- | --- | --- |
|  | **EYO** | **Rate of change** | **p-value** | **Rate of change** | **p-value** | **Rate of change** | **p-value** | **p-value comparing DIAD, EOAM, & EONA** |
| Cognitive Composite, mean (SE) | **1** | -0.20 (0.06) | 0.0005 | -0.39 (0.17) | 0.0219 | -0.29 (0.13) | 0.0274 | 0.2811^1^, 0.5549^2^, 0.6160^3^ |
|  | **5** | -0.36 (0.07) | <.0001 | -0.16 (0.09) | 0.0820 | -0.36 (0.08) | <.0001 | 0.0571^1^, 0.9613^2^, 0.0511^3^ |
|  | **10** | -0.55 (0.16) | 0.0007 | 0.13 (0.26) | 0.6245 | -0.46 (0.21) | 0.0301 | **0.0260^1^**, 0.7203^2^, 0.0737^3^ |
| CDR-SB, mean (SE) | **1** | 0.89 (0.29) | 0.0032 | 1.52 (0.51) | 0.0038 | 1.26 (0.42) | 0.0038 | 0.2623^1^, 0.4567^2^, 0.6775^3^ |
|  | **5** | 2.11 (0.33) | <.0001 | 1.64 (0.30) | <.0001 | 1.53 (0.28) | <.0001 | 0.2252^1^, 0.1225^2^, 0.7502^3^ |
|  | **10** | 3.63 (0.71) | <.0001 | 1.78 (0.79) | 0.0268 | 1.87 (0.70) | 0.0084 | **0.0749^1^, 0.0681^2^**, 0.9270^3^ |
| MMSE, mean (SE) | **1** | -1.33 (0.48) | 0.0071 | -4.10 (0.96) | <.0001 | -3.39 (0.74) | <.0001 | **0.0078^1^, 0.0165^2^**, 0.5453^3^ |
|  | **5** | -3.05 (0.57) | <.0001 | -2.14 (0.59) | 0.0005 | -3.18 (0.49) | <.0001 | 0.2170^1^, 0.8473^2^, 0.1195^3^ |
|  | **10** | -5.21 (1.24) | <.0001 | 0.31 (1.51) | 0.8380 | -2.91 (1.23) | 0.0193 | **0.0047^1^**, 0.1717^2^, 0.0926^3^ |
| NPI-Q, mean (SE) | **1** | 0.33 (0.83) | 0.6952 | 0.67 (1.36) | 0.6216 | 1.78 (1.16) | 0.1305 | 0.8183^1^, 0.2894^2^, 0.5308^3^ |
|  | **5** | 1.62 (1.03) | 0.1184 | 1.17 (0.87) | 0.1807 | 0.29 (0.82) | 0.7199 | 0.7098^1^, 0.2616^2^, 0.3892^3^ |
|  | **10** | 3.22 (2.33) | 0.1681 | 1.78 (2.34) | 0.4479 | -1.56 (2.18) | 0.4751 | 0.6546^1^, 0.1240^2^, 0.2847^3^ |
| Category Fluency (Animals) , mean (SE) | **1** | -0.98 (0.50) | 0.0532 | -0.89 (1.41) | 0.5326 | -1.52 (1.09) | 0.1661 | 0.9459^1^, 0.6375^2^, 0.7117^3^ |
|  | **5** | -1.89 (0.66) | 0.0047 | 0.09 (0.80) | 0.9103 | -1.40 (0.72) | 0.0544 | 0.0352^1^, 0.5515^2^, 0.1066^3^ |
|  | **10** | -3.03 (1.44) | 0.0378 | 1.31 (2.24) | 0.5594 | -1.25 (1.84) | 0.4973 | 0.1054^1^, 0.4260^2^, 0.3697^3^ |
| Category Fluency (Vegetable) , mean (SE) | **1** | -0.66 (0.38) | 0.0879 | -0.91 (1.14) | 0.4239 | -0.41 (0.95) | 0.6676 | 0.8267^1^, 0.7987^2^, 0.7214^3^ |
|  | **5** | -1.45 (0.51) | 0.0054 | -1.21 (0.64) | 0.0609 | -2.04 (0.59) | 0.0008 | 0.7385^1^, 0.3711^2^, 0.2572^3^ |
|  | **10** | -2.44 (1.12) | 0.0315 | -1.57 (1.83) | 0.3912 | -4.08 (1.43) | 0.0052 | 0.6867^1^, 0.3453^2^, 0.2756^3^ |
| Letter Fluency, mean (SE) | **1** | -0.36 (0.38) | 0.3531 | -0.55 (4.09) | 0.8933 | 2.73 (1.44) | 0.0646 | 0.9641^1^, **0.0368^2^**, 0.4478^3^ |
|  | **5** | -1.16 (0.57) | 0.0464 | -0.13 (1.39) | 0.9259 | -1.91 (0.99) | 0.0580 | 0.4798^1^, 0.4415^2^, 0.2713^3^ |
|  | **10** | -2.16 (1.23) | 0.0832 | 0.39 (6.71) | 0.9532 | -7.71 (2.53) | 0.0030 | 0.7080^1^, 0.0428^2^, 0.2592^3^ |
| Logical Memory (Immediate Recall), mean (SE) | **1** | -0.52 (0.33) | 0.1219 | -0.32 (1.15) | 0.7813 | -0.28 (1.15) | 0.8055 | 0.8594^1^, 0.8389^2^, 0.9816^3^ |
|  | **5** | -1.00 (0.44) | 0.0268 | -1.34 (0.76) | 0.0789 | -1.63 (0.68) | 0.0181 | 0.6680^1^, 0.3952^2^, 0.7580^3^ |
|  | **10** | -1.60 (0.95) | 0.0972 | -2.61 (1.93) | 0.1771 | -3.31 (2.07) | 0.1134 | 0.6364^1^, 0.4443^2^, 0.8039^3^ |
| Logical Memory (Delayed Recall), mean (SE) | **1** | -0.16 (0.29) | 0.5795 | 0.36 (1.01) | 0.7261 | -0.40 (1.04) | 0.7030 | 0.6041^1^, 0.8213^2^, 0.5946^3^ |
|  | **5** | -0.74 (0.40) | 0.0655 | -1.81 (0.69) | 0.0100 | -1.84 (0.62) | 0.0040 | 0.1422^1^, 0.1050^2^, 0.9696^3^ |
|  | **10** | -1.46 (0.85) | 0.0900 | -4.51 (1.75) | 0.0110 | -3.64 (1.89) | 0.0580 | 0.1199^1^, 0.2877^2^, 0.7313^3^ |
| Digit Symbol, mean (SE) | **1** | -4.42 (1.40) | 0.0031 | -10.34 (5.45) | 0.0670 | 1.93 (7.56) | 0.7985 | 0.2750^1^, 0.4106^2^, 0.1902^3^ |
|  | **5** | -7.42 (1.94) | 0.0003 | -3.34 (3.41) | 0.3299 | -7.73 (4.24) | 0.0716 | 0.2381^1^, 0.9458^2^, 0.3864^3^ |
|  | **10** | -11.16 (4.09) | 0.0084 | 5.39 (8.43) | 0.5249 | -19.81 (12.58) | 0.1177 | 0.0825^1^, 0.5087^2^, 0.0957^3^ |
| Digit Span Forward, mean (SE) | **1** | -0.38 (0.20) | 0.0608 | -0.80 (0.57) | 0.1690 | -0.40 (0.44) | 0.3658 | 0.4696^1^, 0.9584^2^, 0.5736^3^ |
|  | **5** | -1.50 (0.26) | <.0001 | -0.61 (0.32) | 0.0575 | -0.66 (0.28) | 0.0220 | **0.0181^1^, 0.0108^2^**, 0.8930^3^ |
|  | **10** | -2.91 (0.57) | <.0001 | -0.38 (0.90) | 0.6701 | -0.99 (0.74) | 0.1825 | **0.0190^1^, 0.0317^2^**, 0.5957^3^ |
| Digit Span Backward, mean (SE) | **1** | -0.30 (0.14) | 0.0456 | 0.17 (0.43) | 0.6976 | 0.04 (0.33) | 0.9003 | 0.2785^1^, 0.3134^2^, 0.8066^3^ |
|  | **5** | -0.31 (0.22) | 0.1551 | -0.62 (0.26) | 0.0216 | -0.89 (0.23) | 0.0003 | 0.3379^1^, 0.0371^2^, 0.3892^3^ |
|  | **10** | -0.32 (0.48) | 0.5082 | -1.60 (0.74) | 0.0332 | -2.04 (0.60) | 0.0010 | 0.1537^1^, 0.0221^2^, 0.6363^3^ |
| Trailmaking Test Part A, mean (SE) | **1** | 7.10 (3.90) | 0.0751 | 13.11 (12.32) | 0.2922 | -12.12 (14.86) | 0.4163 | 0.6281^1^, 0.2055^2^, 0.1768^3^ |
|  | **5** | 22.32 (5.10) | <.0001 | 12.04 (6.78) | 0.0800 | 8.19 (8.02) | 0.3099 | 0.1822^1^, 0.0849^2^, 0.6696^3^ |
|  | **10** | 41.36 (11.21) | 0.0004 | 10.71 (19.96) | 0.5933 | 33.58 (22.12) | 0.1316 | 0.1859^1^, 0.7433^2^, 0.4427^3^ |
| Trailmaking Test Part B, mean (SE) | **1** | 18.59 (8.77) | 0.0430 | 19.02 (33.29) | 0.5691 | -97.82 (38.77) | 0.0131 | 0.9899^1^, 0.0036^2^, 0.0177^3^ |
|  | **5** | 21.70 (15.34) | 0.1627 | 33.31 (18.37) | 0.0739 | 31.92 (24.06) | 0.1888 | 0.5757^1^, 0.6798^2^, 0.9570^3^ |
|  | **10** | 25.59 (35.00) | 0.4675 | 51.17 (53.12) | 0.3377 | 194.09 (66.96) | 0.0045 | 0.6839^1^, 0.0224^2^, 0.0915^3^ |
| Boston Naming Test, mean (SE) | **1** | -0.65 (0.53) | 0.2283 | 1.11 (1.97) | 0.5753 | -4.51 (2.09) | 0.0325 | 0.3697^1^, 0.0734^2^, 0.0518^3^ |
|  | **5** | -1.78 (0.67) | 0.0096 | -0.78 (1.18) | 0.5079 | -3.82 (1.03) | 0.0004 | 0.4143^1^, 0.0749^2^, 0.0362^3^ |
|  | **10** | -3.20 (1.41) | 0.0263 | -3.15 (3.04) | 0.3019 | -2.95 (3.27) | 0.3687 | 0.9892^1^, 0.9429^2^, 0.9635^3^ |

Logical Memory, Category Fluency, Letter Fluency, Digit Span, Boston Naming Test and Digit Symbol: lower scores indicating poorer cognitive performance. Trail making Test scores: higher scores indicating poorer cognitive performance.Cognitive Composite is the mean of the standardized scores for Animal naming, delayed recall, Digit Symbol, and MMSE tests. * p-values were adjusted for baseline EYO, sex, education, and APOE4 status. ^1^DIAN vs EOAM; ^2^DIAN vs EONA, ^3^EOAM vs EONA.

**Supplementary Table 4. ﻿ CSF biomarker levels: dominantly inherited Alzheimer’s disease (DIAD) versus early-Onset Alzheimer’s Disease (sEOAD).**

| **﻿Characteristic** | **DIAN Non-mutation carrier^1^**  **N=166** | **DIAD**  **N=91** | **sEOAD**  **N=37** | **Significance Level**  **(p value)** |
| --- | --- | --- | --- | --- |
| AB-42 (pg/ml), mean (SD) | 451.0 (141.6) | 244.9 (117.2) | 298.9 (81.2) | 0.06 |
| Total Tau (pg/ml), mean (SD) | 58.1 (27.1) | 166.1 (97.8) | 145.4 (65.0) | 0.37 |
| pTau-181 (pg/ml), mean (SD) | 29.3 (10.6) | 87.3 (39.3) | 59.7 (24.8) | **0.006** |
| pTau-I81/AB-42, mean (SD) | 0.07(0.03) | 0.4(0.3) | 0.2(0.1) | **<0.001** |

Legend: All DIAN non-mutation carrier were asymptomatic (CDR=0). p-values were adjusted for EYO, CDR, age, and APOE ε4 status.
